# Caregiver Depression Among Home-Bound Stroke Patients in an Urban Community

**DOI:** 10.7759/cureus.17948

**Published:** 2021-09-13

**Authors:** Ozdalifah Omar, Aznida Firzah Abdul Aziz, Mohd Fairuz Ali, Saidatul Ezy Hazika Ali Ja, Md Parvez Eusof Izzudin

**Affiliations:** 1 Department of Family Medicine, Faculty of Medicine, Universiti Kebangsaan Malaysia Medical Centre, Kuala Lumpur, MYS; 2 Klinik Kesihatan Rantau Panjang, Ministry of Health, Malaysia, Kelang, MYS

**Keywords:** cerebrovascular disorders, depression, caregiver, stroke, primary care

## Abstract

Introduction

The sudden undertaking of being a caregiver for a spouse or family member afflicted with a stroke can cause adverse psychological consequences. In Malaysia, the majority of stroke patients return home to be cared for by family members and continue rehabilitation as outpatients. In most local urban communities, the practice of shared caregiving is observed among stroke caregivers either out of necessity or familism. Sole or primary caregivers who share their homes with stroke patients would be more challenged physically and psychologically compared to secondary or joint caregivers. Sharing the caregiving responsibilities is believed to lighten the burden on primary caregivers. This study aims to determine the proportion and associated factors of depression among urban-dwelling caregivers of home-bound stroke patients receiving long-term care from a university-based primary care clinic.

Methodology

A cross-sectional study involving 123 primary and secondary caregivers of stroke patients was conducted at Klinik Primer PPUKM Cheras (KPPC) and the outpatient Medical Rehabilitation Services Department Hospital Canselor Tuanku Muhriz (HCTM), Cheras Kuala Lumpur. A self-administered questionnaire comprising of sociodemographic characteristics, the Beck Depression Inventory-II (BDI-II), and the Multidimensional Scale of Perceived Social Support questionnaire (MSPSS) was used. The functional status of the stroke patients was assessed using the Modified Rankin Score (MRS).

Results

The proportion of respondents with depression was 20.3% (n=25). Depression was associated with caregivers’ age (CI=42.23-50.09, p=0.016), presence of illness (p=0.001), and being a sole caregiver (p=0.001). There is also an association found between caregiver depression with longer duration post-stroke (CI= 12.75-16.13, p<0.001), longer time spent for caregiving (CI= 117.73-135.87, p=0.004), and more functionally dependant patients (p=0.002).

Conclusion

Depression affects one in five caregivers of home-bound stroke patients residing in the urban community. The primary care provider should be more vigilant in screening for depression, especially among caregivers who are from older age groups, have ongoing health problems, and are sole caregivers to patients who are functionally dependant.

## Introduction

Stroke is the third cause of mortality in Malaysia and it remains one of the most disabling neurological conditions [1]. Caregivers play an important role in helping the stroke patient adapt to the disability and achieve his or her possible maximum functional potential. The caregiver role is usually entrusted to family members, typically the spouse [2]. This pattern is also observed in Malaysia, whereby family members provide the most assistance for stroke survivors, either during hospitalization or at home [3]. Caregiving duties are often shared among family members, i.e., spouse or offspring of home-bound stroke patients. The caregiving process for stroke patients has been proven to be associated with a psychological burden to the caregiver [4], which may manifest as emotional disturbances and mental health illnesses. According to the Family Caregiver Alliance, "family caregivers experience high rates of depression, stress, and other mental health problems" [5]. As shown in a study conducted by Loh in Singapore, a high prevalence of depressive and anxiety symptoms were found among caregivers of stroke survivors, which were 40.2% and 21.4%, respectively [2]. The greatest concern with depression in these groups of caregivers is the deleterious effect it has on both their own and the patient’s quality of life (QOL). The caregiver’s QOL can be affected in terms of physical, psychological, social, and environmental [6]. Meanwhile, the QOL of the stroke patients is found to be affected by the caregiver’s depressive symptoms in the context of social participation, mood, and emotion [7].

There are many factors associated with depression among caregivers of stroke patients that have been well studied in various countries. However, despite knowing the burden, associated factors, and effects of depression in this group of caregivers, local data on caregivers of stroke survivors is scarce, especially in those co-habiting with care recipients in the community. 

The dearth of local data on these caregivers complicates the development of a support or respite program for this group. This is because there is insufficient evidence to support the need for such programs. Thus, this study aims to provide data on the proportion of adverse psychological outcomes, namely depression, among caregivers of stroke patients in our local population. It is hoped that the identification of caregivers with a higher risk of developing psychological distress may help facilitate planning for respite care services and support programs for caregivers of stroke patients in Malaysia, in an attempt to improve the overall health of both caregivers and care recipients.

## Materials and methods

A cross-sectional study was conducted at the Klinik Primer PPUKM Cheras (KPPC) and the outpatient Medical Rehabilitation Services Department Hospital Canselor Tuanku Muhriz (HCTM), Cheras Kuala Lumpur; both of which are located in an urban setting. The study was conducted between January and April 2018. KPPC is a university-based primary care clinic that provides a dedicated long-term stroke care service (Klinik Lanjutan Strok, KLS). The patients who receive treatment at KLS normally will undergo outpatient stroke rehabilitation at the Medical Rehabilitation Services Unit of Hospital Canselor Tuanku Muhriz (HCTM), a tertiary teaching hospital located one kilometer apart.

All accompanying caregivers of stroke patients undergoing follow-up at the KLS and patients attending outpatient physiotherapy or occupational therapy sessions at Rehabilitation Services Unit HCTM were invited to participate in this study. The inclusion criteria were caregivers of stroke patients aged 18 years and above and able to read and write in Malay or English language. Caregivers who were illiterate, diagnosed or undergoing treatment for depression, and caregivers of stroke patients residing in nursing homes were excluded.

As there is no known prevalence of stroke or stroke caregivers in Malaysia, the Krejcie and Morgan formula was used to determine the sample size required, using the KLS patient registry. The study was limited to one caregiver per stroke patient. A total of 135 respondents were approached, but two were excluded as they did not fulfill the inclusion criteria. Another 10 respondents were excluded as they did not complete the questionnaire. The final number of respondents included in this study were 123, which makes the response rate 91.1%.

A self-administered assisted questionnaire was given to all participants who consented to participate in this study. Minimal assistance was provided by the researcher upon request, i.e., mainly to provide assistance in visually challenged respondents. The questionnaire provided was either in English or Malay language based on participant’s preferences and consisted of four sections. A pilot study was conducted involving 10 respondents each from both the study sites beforehand to ensure that the respondents could comprehend the study tools used.

The first section was on the sociodemographic details, the status of the caregiver of being a sole or joint caregiver, duration post-stroke of the patients cared, and the total time spent for caregiving purposes in minutes in a day. Duration post-stroke was calculated from the date of the last stroke until the date the respondent was recruited. In the event that the exact date of the last stroke is unknown or could not be verified, the date of stroke is taken as the first day of the particular month of the year where the stroke was the last known to have occurred. Total caregiving time is calculated in minutes over a day and includes all activities that involved direct physical contact such as feeding, bathing, toileting, and physical therapy. The questionnaires were triangulated based on a literature review on previous studies on the mental health status of caregivers of stroke patients and underwent face validation with a panel of experts consisting of family medicine specialists who provided longer-term care stroke services.

The Beck Depression Inventory-II (BDI-II) and its validated Malay version were used to assess for depression [8]. The respondents were then classified based on the total scores, into no depression (score 0-10), mild depression (score 11-20), moderate depression (score 21-30), and severe depression (score over 31). The social support of the respondents was assessed using the English and validated Malay version of the Multidimensional Scale of Perceived Social Support questionnaire (MSPSS) [9-12]. It consisted of subsections to assess the perceived social support from the respondent’s significant other, family and friends. The scoring is then scored into the average for Significant Other subscales, Family subscale, Friends subscale, and Total social support. The respondents were then classified into having perceived poor social support (scores 1-2.9), perceived moderate social support (scores 3-5), and perceived high social support (scores 5.1-7). The final section of the questionnaire was the assessment of the functional status or independence level of the stroke patient by the researcher using the Modified Rankin Scale (MRS) [13]. The MRS classifies stroke patients into six subgroups, ranging from 0 (no symptoms) to 6 (dead). However, in view of the subject of this research involves stroke patients under follow-up or therapy, the score of 6 (dead) was omitted and the last score considered was 5 (severe disability, bedridden, incontinent, and requiring constant nursing care and attention). The scores were further sub-classified into functionally independent (scores 0-2) and functionally dependent (scores 3-5).

All data were analysed using the Statistical Package for Social Sciences (SPSS), version 23 (IBM Corp., Armonk, NY). The profile of the respondents, duration post-stroke, time taken (i.e., direct physical contact) in a day for caregiving, the prevalence of depression, MSPSS score, and MRS score were described using descriptive analysis. The relationship between the sociodemographic details, status of caregiver (sole or joint), duration post-stroke, time taken in a day for caregiving, MSPSS and MRS scores with depression among the stroke caregivers were analysed using either independent T-test, chi-square, Fisher’s exact test or the Mann Whitney U test depending on the skewness of the data. The crude and adjusted OR, together with 95% confidence interval (CI) were used to define the strength of the associations. The p-value of <0.05 indicates a statistically significant association.

All respondents who were found positive for depression based on the BDI-II were contacted and alerted to the team leader of the KLS for further assessment and management.

## Results

The median (interquartile range, IQR) age of the respondents was 38 (24) years old. The majority of the respondents were female (61.0%), Malay (61.0%), had no medical illnesses (75.6%), and joint caregivers (64.2%). The majority (46.3%) of the caregivers were children of stroke patients (Table 1).

**Table 1:** Sociodemographic characteristics of caregivers of urban home-bound stroke patients

Variables	Mean (SD)	Median (IQR)	n = 123 (%)
Caregiver’s age (years)		38 (24)	
<29			45 (36.6)
30–39			18 (14.6)
40–49			20 (16.3)
50–59			36 (29.3)
>60			4 (3.2)
Gender			
Male			48 (39.0)
Female			75 (61.0)
Ethnicity			
Malay			75 (61.0)
Chinese			34 (27.6)
Indian			13 (10.6)
Others			1 (0.8)
Presence of illness			
Yes			30 (24.4)
No			93 (75.6)
Relationship with patient			
Spouse			48 (39.0)
Children			57 (46.3)
Siblings			6 (4.9)
Other family members			12 (9.8)
Education level			
Up to secondary			62 (50.4)
Tertiary			61 (49.6)
Income (RM)	3104.1 (SD 2069.2)		
Status of caregiver			
Sole			45 (35.8)
Joint			79 (64.2)

The median (IQR) duration post-stroke was 10(9) months, with the minimum duration being two months and a maximum duration of 24 months. Total caregiving time in a day involves all caregiving activities that have direct caregiver-patient contacts such as feeding, bathing and toileting, physical therapy, and time taken for outpatient follow-up visits. Other activities that do not involve caregiver-patient contact such as time taken by the respondent to buy patient’s necessities and doing laundry of patient’s clothes were not included. The median (IQR) time spent for caregiving in a day was 90 (35) minutes. The minimum time taken for caregiving in a day was 60 minutes and the maximum was 235 minutes.

In terms of social support, the majority (63.4%) of caregivers perceived that they received moderate support while 35.8% perceived high support from significant others, family, and friends.

Based on the functional status of the care recipients, 45.5% scored 3 on the MRS scale, which meant that the patients had a moderate disability requiring some help, but they are able to walk without assistance. Further classification of the MRS indicates that the majority (78%, 96/123) of the patients were functionally dependant (MRS 0-2) on their caregivers (Table 2). This is expected as the patients were still under stroke during clinic follow-up and needing rehabilitation therapy.

**Table 2:** Functional status/independence level of the home-bound stroke patients

MRS score	Functional ability description	n (%)
0	No symptoms at all	2 (1.6)
1	No significant disability despite symptoms; able to carry out usual duties and activities	3 (2.4)
2	Slight disability: unable to perform all previous activities but able to look after own affairs without assistance	22 (17.9)
3	Moderate disability: requiring some help but able to walk without assistance	56 (45.5)
4	Moderately severe disability: unable to walk without assistance and unable to attend own bodily needs without assistance	34 (27.6)
5	Severe disability: bedridden, incontinent, and requiring constant nursing care and attention	6 (5.0)

Using the BDI-II, 20.3% (25/123) respondents were found to have depression in this study. Out of the 25, slightly more than half (56.0%, 14/25) had mild depression, followed by moderate depression (9/25) and severe depression (2/25).

From univariate analysis, six factors were found to be associated with depression among the respondents; caregivers’ age (p=0.005), presence of illness (p<0.001), whether sole or joint caregiver (p=0.002), duration post-stroke (in months; p<0.001), total caregiving time in a day (in minutes; p<0.001), and the functional status of the stroke patient (p<0.001; Table 3).

**Table 3:** Factors associated with depression among caregivers of home-bound stroke patients

Variable	Depression		p-Value
	No n = 98 (%)	Yes n = 25 (%)	
Caregiver’s age median (IQR)	34.5 (24)	50(14)	0.005^#^
<29	43 (95.6)	2 (44.4)	
30–39	14 (77.8)	4 (22.2)	
40–49	14 (92.2)	6 (7.8)	
50–59	23 (63.9)	13 (36.1)	
>60	4(100.0)	0 (0.0)	
Gender			
Male	41 (85.4)	7 (14.6)	
Female	57 (76.0)	18 (24.0)	
Ethnicity			0.623
Malay	62 (82.7)	13 (17.3)	
Chinese	26 (76.5)	8 (23.5)	
Indian	9 (69.2)	4 (30.8)	
Others	1 (100.0)	0 (0.0)	
Presence of illness			<0.001^*^
Yes	17 (56.7)	13 (43.3)	
No	81 (87.1)	12 (12.9)	
Relationship with patient			0.061
Spouse	32 (66.7)	16 (33.3)	
Children	49 (86.0)	9 (14.0)	
Siblings	6 (100.0)	0 (0.0)	
Other family members	11 (91.7)	1 (8.3)	
Education level			0.059
Up to secondary	44 (71.0)	18 (29.0)	
Tertiary	54 (88.5)	7 (11.5)	
Income (RM)	3223.5 (SD 2166.0)	2636.0 (SD 1586.7)	0.206
Status of caregiver			0.002^*^
Sole	28 (63.6)	16 (36.4)	
Joint	70 (88.6)	9 (11.4)	
Duration post-stroke (in months) median (IQR)	9.0 (6.0)	16.0 (5.0)	<0.001^#^
Total caregiving time/day (minutes)	90.0 (SD 20.0)	120 (SD 13.0)	<0.001^#^
Social support			0.434
Low	0 (0.0)		
Moderate	55 (70.5)		
High	43 (97.7)		
Functional status (MRS)			<0.001^+^
Total score	2.8 (SD 0.8)	4.1 (SD 0.6)	
Functionally independent (n%)	27 (100.0)	0 (0.0)	
Functionally dependent (n%)	71 (74.0)	25 (26.0)	

The older the age of the respondent, there is a higher the association with depression, as well as those with medical illnesses. Sole caregivers are found to be depressed, as compared to those who jointly care for the patient with assistance from others. Respondents taking care of stroke patients with a longer duration post-stroke and respondents who spent a longer duration in a day for caregiving purposes are also associated with depression. The respondents caring for functionally dependant stroke patients have a likelihood of depression (Table 3).

## Discussion

This study provides the proportion of depression among caregivers of home-bound urban stroke patients, data that have not been provided in any other study in Malaysia to date. Compared with the global prevalence of depression among caregivers of stroke patients conducted abroad, the percentage of caregivers with depression found in this study at 20.3% is relatively low [2]. The apparent disparity in the results might be contributed by the difference in the study settings. The previous studies were conducted in countries that have a significantly different sociodemographic pattern, particularly with regard to ethnicity. At the time this study was conducted, there were no other similar studies looking specifically for depression among stroke patients’ caregivers carried out in countries where the population share almost similar ethnic distribution as Malaysia; such as Indonesia, Singapore, or Thailand. This difference in ethnicity distribution provides different cultural values in terms of familism and coping mechanisms [13,14]. Familism is a cultural value that refers to strong identification and solidarity of individuals with their family and strong normative feelings of loyalty, dedication, reciprocity, and attachment to both nuclear and extended family members [14]. This is in stark contrast to the practice of individualism among the caregivers in Western countries where most of the previous studies were conducted. This concept of familism and filial piety plays an important role in terms of the coping mechanism of the caregivers who are taking care of their family members with stroke. It is theorized that this may contribute to better coping mechanisms among the caregivers, hence the lower depression rates. This theory, however, needs further studies to be conducted among Asian, particularly South-East Asian countries for it to be accepted.

This is the first time that the BDI-II is used in the primary care setting in Malaysia, specifically among caregivers of stroke patients. The Patient Health Questionnaire-9 (PHQ-9) is more commonly used to screen for depression in the primary care settings in this country. Both BDI-II and PHQ-9 have psychometric properties that are generally strong in terms of internal consistency, factor structure, and convergent validity [15]. BDI-II was chosen as it is shown to have better reliability in terms of internal consistency (Cronbach α 0.92) than PHQ-9 (Cronbach α 0.89) [16]. Furthermore, the BDI-II has better sensitivity (94%) and specificity (92%) as compared to the PHQ-9 (sensitivity 87%, specificity 81%) when used among the general population, and in this case, being caregivers of the stroke patients [17].

Although there are studies looking into the differences in depression between single and married caregivers of stroke patients [7,18,19], there are no studies to date which address differences between the sole versus joint caregiving factor. Joint caregiving practices are a common observation among caregivers of long-term home-bound stroke patients attending the KLS, particularly among stroke patients with large families. Family members contribute to caregiving by helping to provide either financial assistance, transportation or even accompanying patients for follow-up appointments or outpatient rehabilitation sessions. We postulate that as a consequence of this shared caregiving arrangement, joint caregivers provide a short respite period for the primary caregiver when the patient attends outpatient clinics or rehabilitation sessions. This study has found an association that indicates the higher likelihood of depression among sole caregivers. Although various theories can be postulated based on this finding; such as caregiver fatigue due to carrying various roles in caregiving as well as a heavier burden on a single person, further research is warranted to find the exact cause for the finding of higher depression rates among sole caregivers in this study.

This study quantifies total caregiving time spent by caregivers of stroke patients for caregiving purposes. The significant association between caregiving time and depression correlates with findings in this study which show that the majority of the stroke patients were functionally dependant, thus required more caring time. The reason for the significant correlation may be due to fatigue and reduced quality of life of the caregiver due to longer duration spent on care provision for the patient [20]. However, these findings require further in-depth studies to determine the possible reasons for depression among those who spend more time caregiving for stroke patients.

This study's findings concur with earlier studies that showed caregivers of more functionally dependant stroke patients tend to be depressed [21]. The majority of the stroke patients in this study were functionally dependant, which explains why they are still on frequent follow-up and rehabilitation.

Limitations and strength

First, the sample size was limited to those who had access to the two study sites, thus there may be a core number of caregivers that may have been missed. Second, homogeneity of stroke patients (i.e., stage of stroke recovery) with regards to stroke caregivers’ profile could not be addressed as there is no standardised transfer of care beyond the acute stroke period. Patients particularly at public hospitals are mostly discharged to the nearest public primary care health centres or followed up under specialist outpatient clinics in the tertiary hospitals.

The number of caregivers who were positive for depression was small, thus preventing further statistical analysis such as multilinear regression. Thus, compounding factors that may contribute to the significant associated factors found with depression could not be excluded.

Furthermore, this study was conducted in an urban community, hence the findings may not be generalized to the other communities, particularly to the semi-urban or rural communities.

Nevertheless, to date, this study is the first to determine the proportion of depression and the associated factors among caregivers of stroke patients residing at home, in Malaysia. This study has also looked at the status of sole versus joint caregiver, duration post-stroke, and total caregiving time and its effect on caregivers.

Recommendations

Further qualitative research should be conducted in the future to explore the reasons for depression. This may help to identify further factors that may have not been identified in this or previous studies (e.g., unmet needs of the stroke caregiver). Multicentre studies involving public primary healthcare facilities with randomised sampling and a larger sample size should be conducted as it may allow the exclusion of compounding factors, as well as provide a better representation of the Malaysian population.

## Conclusions

In summary, depression among caregivers of urban home-bound stroke patients affects every one in five caregivers, with the majority having mild depression. Primary care providers should anticipate caregivers’ depression among those providing care for home-bound stroke patients who are older, with a background of personal medical illnesses, sole caregivers, i.e., caring for patients on his/her own, care recipients with longer post-stroke duration, spending more time for direct contact caregiving, and among those caring for functionally dependant patients.
